# Dr. Alexander Thompson Bull

**Published:** 1910-09

**Authors:** 


Dr. Alexander Thompson Bull, of Buffalo, died in his apartments at the Iroquois Hotel, August 15, 1910, aged 83 years. About ten days before his death he misstepped in boarding a street car, sustaining shock from which he did not recover because of his age. Dr. Bull came to Buffalo from Canada in 1864, and soon became one of the leading homeopathic physicians in this city, occupying a high place in the esteem of his confreres until his death. He retired from practice several years ago, but his was a familiar figure on the streets where he was seen almost every day until injured as above related. Dr. Bull enjoyed the respect of the medical profession outside the lines of his own sectarian affiliation, so much so, indeed, that he recently was elected to honorary life membership in the Medical Society of the County of Erie and in the Buffalo Academy of Medicine.

